# Efficacy and Safety of Regorafenib Combined with Toripalimab in the Third-Line and beyond Treatment of Advanced Colorectal Cancer

**DOI:** 10.1155/2021/9959946

**Published:** 2021-09-24

**Authors:** Wei Yu, Qiaomeng Tao, Yufeng Zhang, Fengming Yi, Long Feng

**Affiliations:** ^1^Department of Oncology, Second Affiliated Hospital of Nanchang University, Nanchang 330006, China; ^2^JiangXi Key Laboratory of Clinical and Translational Cancer Research, Nanchang 330006, China

## Abstract

**Background:**

The most effective treatment of immune checkpoint inhibitors (ICIs) is restricted in microsatellite instability (MSI-H) subsets of advanced colorectal cancer, but MSI-H only accounts for 4-5% among them. ICIs are completely ineffective in advanced colorectal cancer patients with microsatellite stable (MSS), according to literatures published. Regorafenib is a novel tyrosine kinase inhibitor (TKIs) that could normalize tumor blood vessels by inhibiting vascular endothelial growth factor receptor and its downstream, thus improving cytotoxic T cell infiltration in tumor microenvironment, which has a synergistic effect with ICIs. Toripalimab is a type of anti-PD-1 monoclonal antibody produced by Junshi Biosciences in China. Herein, we aimed to explore the efficacy and safety of regorafenib combined with toripalimab in the third-line and beyond treatment of advanced colorectal cancer.

**Methods:**

We evaluated the outcomes of MSS patients with advanced colorectal cancer who received regorafenib combined with toripalimab in the Second Affiliated Hospital of Nanchang University from June 2019 to January 2021. These patients had previously received at least second-line treatment; the regimens were oxaliplatin and irinotecan-based chemotherapy and/or accompanied with bevacizumab or cetuximab. Thirty-three patients were treated orally with regorafenib 80 mg or 120 mg once daily for 21 days, 28 days as a cycle, combined with intravenous toripalimab until disease progression or intolerant to adverse reactions. We used the Kaplan–Meier method to estimate the rate of progression-free survival (PFS) and log-rank method to do a statistical test of the survival curve. The Cox regression model was used to analyze the influence of multiple factors on PFS. The primary endpoints were objective remission rate (ORR) and disease control rate (DCR). The secondary endpoints were the incidence of adverse reactions and median progression-free survival (mPFS).

**Results:**

The evaluation of treatment effects was assessed according to RECIST 1.1. Four patients (12.12%) got partial response, twelve patients (36.36%) experienced stable disease, and seventeen patients (51.52%) suffered progressive disease. ORR was 12.12% and DCR was 48.48%. mPFS was 113 days (95% CI: 0–272.1). In univariate analysis, patients who had previously received second-line treatment were significantly better than those who had received third-line or more treatment (*p*=0.005). Lung metastasis was a negative factor in combined therapy (*p*=0.032). Five patients without previous treatment of bevacizumab were effective. Previous treatment without bevacizumab showed a trend of effective when combination therapy (*p*=0.034). It was also a positive factor that the Eastern Cooperative Oncology Group performance status (ECOG) score was 0 (*p*=0.034). Multivariable Cox regression analysis showed the number of previous chemotherapy lines and excision of primary lesions were independent prognostic factors. The most common treatment-related adverse reactions were hand-foot syndrome (33.33%), liver dysfunction (27.27), hypothyroidism (24.24%), fever (24.24%), fatigue (21.21%), leukopenia (15.15%), hypertension (12.12%), platelet count decreased (6.06%), diarrhea (3.03%), and myocarditis (3.03%); one patient stopped treatment as myocarditis. The incidence of grade 3/4 adverse reactions was 9.09%.

**Conclusions:**

Regorafenib combined with toripalimab has a promising effect in the third-line and beyond treatment of advanced colorectal cancer. In the early use of combination therapy, excision of primary lesions can have a positive impact in regorafenib and toripalimab combination. This treatment-related adverse reactions are tolerant in combined therapy.

## 1. Introduction

The incidence and mortality rates of colorectal cancer are on the rise globally, which ranked third in terms of morbidity and mortality, whatever male or female [1]. With the application of targeted therapies such as bevacizumab or cetuximab combined with chemotherapy, the median overall survival (mOS) of patients with advanced colorectal cancer has already exceeded 30 months [2]. However, the prognosis of patients after the progress of second-line treatment is still poor. Regorafenib is currently the standard third-line treatment drug for advanced colorectal cancer [3, 4], but its efficacy is still not satisfactory.

Effective immunotherapy in colorectal patients mainly exists in the tumor subsets of microsatellite instability (MSI-H) [5], which may be due to the presence of high density infiltrating CD8^+^T cells in MSI-H colorectal cancer tissues, leading to high levels of neoantigens and corresponding high immunogenicity [6]. However, the microsatellite status of colorectal cancer changes dynamically with the progression of cancer. The later the stage is, the lower the proportion of MSI-H patients is. MSI-H cases only account for 4-5% of patients with advanced colorectal cancer [7]. Microsatellite stable (MSS) colorectal cancers may resist to immune checkpoint inhibitors (ICIs) due to a lack of CD8^+^T cells infiltration. Toripalimab is a type of anti-PD-1 monoclonal antibody produced by Junshi Biosciences approved by NMPA in China, and the efficacy is verified by numerous studies [8].

Regorafenib is a new type of small molecule tyrosine kinase inhibitor [9]. It can reduce the regulatory T cell immune inhibitory effect by inhibiting vascular endothelial growth factor receptor. It also can restrain colony-stimulating factor 1 receptor and inhibit the formation of tumor-associated macrophages, which could remove the immunosuppressive effect of the tumor microenvironment [10]. Numerous investigations are trying to explore the potential of combination immunotherapies to convert MSS colorectal cancer to an immune-responsive malignancy [11, 12]. This study aims to explore the efficacy and safety of regorafenib combined with toripalimab in the third-line and beyond the treatment of advanced colorectal cancer to verify the novel combination for future clinical treatment.

## 2. Materials and Methods

### 2.1. General Information

We evaluated the outcomes of MSS patients with advanced colorectal cancer who received regorafenib combined with toripalimab in the Second Affiliated Hospital of Nanchang University from June 2019 to January 2021. These patients had previously received at least second-line treatment. The regimens are oxaliplatin and irinotecan-based chemotherapy and/or combined with bevacizumab or cetuximab. A total of 33 patients with MSS colorectal cancer (18 women and 15 men) were evaluated. The inclusion criteria for the research were as follows. (1) Pathology confirmed colorectal cancer. (2) The age ranks from18 to 80. (3) Patients who had received at least two lines of previous treatment for metastatic colorectal cancer, including oxaliplatin and irinotecan. Prior treatment may include bevacizumab or cetuximab but is not required. (4) Eastern Cooperative Oncology Group (ECOG) physical status score restricted to 0-1. (5) Informed consent has been signed. Exclusion criteria included the following. (1) A history of use of regorafenib. (2) Severe liver and kidney dysfunction and serious underlying diseases. (3) The history of activity or medical history of chronic or recurrent autoimmune diseases. (4) Baseline lesions cannot be measured by the Response Evaluation Criteria in Solid Tumors 1.1 (RECIST1.1). This study was approved by the Ethics Committee of the Second Affiliated Hospital of Nanchang University.

### 2.2. Treatment

Patients were treated orally with regorafenib 80 mg or 120 mg once daily for 21 days, 28 days as a cycle, combined with intravenous toripalimab 240 mg, 21 days as a cycle until disease progression or intolerant to adverse reactions.

### 2.3. Assessment

The patients were examined by computed tomography every eight weeks until disease progression or before follow-up treatment. And the efficacy was evaluated according to RECIST 1.1. Objective response rate (ORR) was defined as the percentage of patients' treatment results that reached complete response (CR) or partial response (PR). Disease control rate (DCR) was defined as the proportion of patients with CR, PR, or stable disease (SD). Progression-free survival (PFS) was defined as the time from the date of combined treatment until the date of disease progression or the date of death as a result of any reason. Various treatment responses were evaluated through independent evaluation by the center of our hospital. Adverse reactions were evaluated according to Common Terminology Criteria for Adverse Events 4.0 (CTCAE4.0).

### 2.4. Statistical Analysis

We used SPSS26.0 to process and analyze the statistics. We used the Kaplan–Meier method to estimate the rate of progression-free survival (PFS) and the log-rank method to do a statistical test of the survival curve. The Cox regression model was used to analyze the influence of multiple factors on PFS.

## 3. Results

### 3.1. Patient Characteristics

A total of 33 patients with advanced colorectal cancer with MSS were included in this study, including 15 males and 18 females, with an average age of 53.64 years old. Twenty-three patients had left-sided colorectal cancer, ten patients had right-sided colon cancer, the primary sites of the 29 patients were resected, and four patients took no resection of the primary lesions. Twenty patients had a history of liver metastasis, twenty-eight patients had a history of lung metastasis, and 22 patients had a history of lymph node metastasis. All patients previously received oxaliplatin, irinotecan, and fluorouracil-based chemotherapy. Twenty-eight patients were treated with bevacizumab, 11 patients were treated with cetuximab, and 12 patients were treated with raltitrexed. There were 16 patients who had received only second-line treatment previously and 17 patients who had received third-line treatment or above. Twenty-one patients underwent gene detection, and eight patients had a KRAS gene mutation. Twenty-three patients received 80 mg of regorafenib. The patient characteristics are given in Table 1.

### 3.2. Efficacy

The evaluations of the treatment effect were according to RECIST1.1. When patients were evaluated as CR, PR, or SD, we considered combination therapy effective.

**Figure 1:**
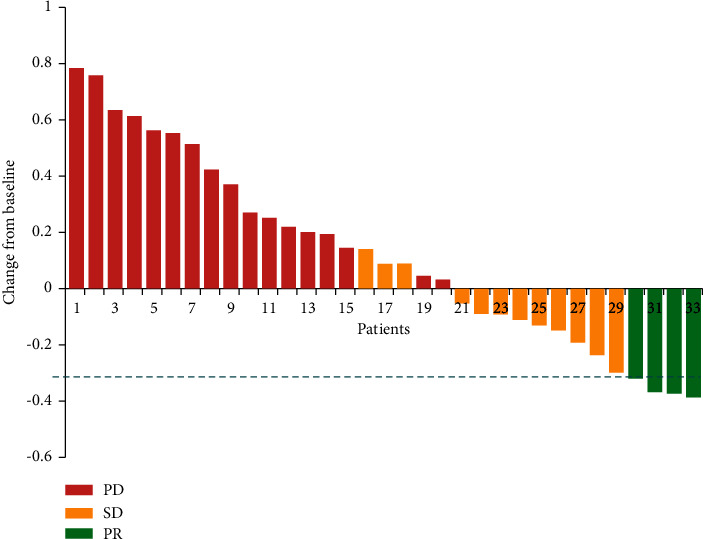
The tumor shrinkage from baseline.

**Figure 2:**
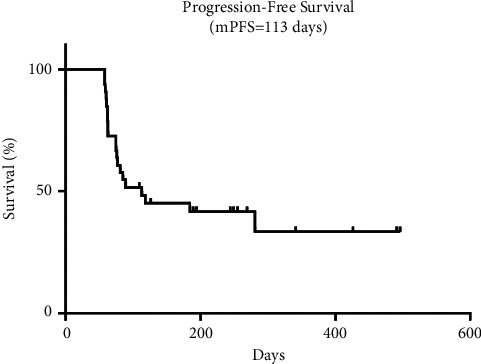
The median progression-free survival of the combined treatment.

Patients who were evaluated as PD was deemed ineffective. Four patients (12.12%) got partial response, twelve patients (36.36%) experienced stable disease, and seventeen patients (51.52%) suffered progressive disease. ORR was 12.12% and DCR was 48.48%. mPFS was 113 days (95% CI: 0–272.1). Eleven patients died and twenty-two patients survived at the end of follow-up. More than half of the patients were alive, so that we could not get mOS for patients. The tumor shrinkage from baseline is shown in Figure 1. The efficacy is given in Table 2 and mPFS is shown in Figure 2.

### 3.3. Univariate Analysis

In univariate analysis, patients who had previously received second-line treatment were significantly better than those who had received third-line or more treatment (*p*=0.005). Lung metastasis was a negative factor in combined therapy (*p*=0.032). Five patients without previous treatment of bevacizumab were effective, and previous therapy without bevacizumab showed a trend of effectiveness when combination therapy (*p*=0.034). It was also a positive factor that Eastern Cooperative Oncology Group performance status (ECOG) score was 0 (*p*=0.034). There were no significant differences in effectiveness in gender, age, liver metastasis, lymph node metastasis, previous cetuximab used, and regorafenib dose (*p* > 0.05). The results are given in Table 3.

### 3.4. Multivariate Analysis

In clinical practice, the location of the primary lesion and surgical resection are related to patient survival. Based on the results of univariate analysis, factors such as the ECOG score, number of previous chemotherapy lines, primary lesion resection, primary lesion location, bevacizumab used, and lung metastasis were included in Cox multivariate regression analysis. The results showed that the number of previous chemotherapy lines and whether the primary lesion was resected were independent prognostic factors of PFS. Early use of regorafenib in combination with toripalimab after second-line treatment in patients with advanced colorectal cancer has a greater survival benefit. Patients with unresected primary lesions had poor efficacy using combination therapy. The results are given in Table 4.

### 3.5. Safety

The most common treatment-related adverse reactions were hand-foot syndrome (33.33%), liver dysfunction (27.27), hypothyroidism (24.24%), fever (24.24%), fatigue (21.21%), leukopenia (15.15%), hypertension (12.12%), platelet count decreased (6.06%), diarrhea (3.03%), and myocarditis (3.03%); one patient stopped treatment as myocarditis. Most patients had mild reactions, which can be continued after symptomatic treatment. The incidence of grade 3/4 adverse reactions was 9.09%. The results are given in Table 5.

## 4. Discussion

Immunotherapy activates the immune system to fight against tumors. The most popular immunotherapy drugs are ICIs in colorectal cancer. KEYNOTE-177 showed that the pabolizumab group was significantly better than the standard first-line treatment group in PFS and ORR (PFS16.5 m vs. 8.2 m; ORR 43.8% vs. 33.1%) in MSI-H advanced colorectal cancer patients [5]. CheckMate 142 enrolled MSI-H advanced colorectal cancer patients who had previously received oxaliplatin or irinotecan-based chemotherapy. The study demonstrated perfect safety and significant clinical efficacy in patients who were treated with nivolumab combined with ipilimumab. ORR was 55%; DCR was 80% for 12 weeks. The effectiveness was independent of KRAS/BRAF mutation status and PD-L1 expression of tissues [13]. These results suggest that immunotherapy may improve the outcome of patients with MSI-H colorectal cancer.

In recent years, regorafenib is one of the third-line treatment regimens for patients with advanced colorectal cancer, which acts in antiangiogenesis, regulation of tumor microenvironment, and inhibition of tumor cell proliferation [9]. However, the curative effect of regorafenib was not satisfactory in some patients. In the MSS preclinical model of colorectal cancer, regorafenib can reduce the infiltration of immunosuppressive macrophages and inhibit the growth of tumor cells with synergistic ICIs [10]. Some studies have shown that antiangiogenesis drugs could reduce the activity of regulatory T cells and reshape the tumor microenvironment. Blocking vascular endothelial growth factors could inhibit mature dendritic cells, making T cells infiltrate into the tumor and cytotoxic T cells more effective and activated. The combination of antiangiogenesis drugs and ICIs could enhance the antitumor function of cytotoxic T cells [14].

The REGONIVO study was a combination of regorafenib and nivolumab in treating advanced colorectal cancer and gastric cancer patients. The dose range of regorafenib was 80–120 mg, and the safety was relatively high. In the colorectal cancer cohort, the ORR of 24 MSS patients with advanced colorectal cancer was 33%, median PFS was 7.9 months, and median OS was not reached. The 1-year survival rate was 68.0%. It was fully demonstrated that the survival benefit of regorafenib combined with nivolumab was much higher than that of the previous standard third-line treatment regimen for colorectal cancer [12]. REGOTORI from China explored the efficacy and safety of regorafenib combined with toripalimab in refractory MSS colorectal cancer. The OS of 39 patients was 15.5 months, and the median duration of response (DOR) was 9.6 months, suggesting that the antitumor efficacy of the regimen was long-lasting. ORR was up to 30% in patients without liver metastasis [15]. REGOMUNE was a study on the combination of avelumab and regorafenib treating third-line and above MSS advanced colorectal cancer. The results showed that the PFS and OS of the patients were similar to those of the previous regorafenib monotherapy, with the median PFS of 3.6 months, the median OS of 10.8 months, and the ORR was 0 [16]. These results suggest that although PD-L1 inhibitors and PD-1 inhibitors act on the PD-1/PD-L1 pathway, there are still differences in antitumor activity.

In the present study, four patients (12.12%) got partial response, twelve patients (36.36%) experienced stable disease, and seventeen patients (51.52%) suffered progressive disease. ORR was 12.12%. DCR was 48.48%. mPFS was 113 days (95%CI: 0–272.1), and median OS was not reached. Five patients without lung metastasis were effective for treatment (*p*=0.032). It is contrary to the REGONIVO, which showed a better outcome in patients with lung metastasis [12]. Patients who had received only standard second-line treatment in the past had better efficacy (*p*=0.005). For the patients with advanced second-line treatment, the combination treatment as early as possible is more beneficial to their survival. Three patients who had not been treated with bevacizumab were effective. Two of them obtained PR (*p*=0.034). Bevacizumab combined with ICIs improved survival in patients with advanced liver cancer [17]. It was also a positive factor that the ECOG score was 0 (*p* = 0.034). Among them, twenty-one patients underwent gene detection, and eight patients had KRAS mutation, with no statistically significant difference in PFS (*p*=0.203). There are some limitations in this study. The sample size was small, and the number of patients with KRAS mutation was lack. So, it was not enough to draw a clear conclusion. To elucidate the impact of these clinical factors on combination therapy outcomes, additional analyses with larger sample sizes are necessary.

Cox multivariate regression analysis showed that the number of previous chemotherapy lines and whether the primary lesion was resected were independent prognostic factors of PFS. Early use of regorafenib in combination with toripalimab after second-line treatment in patients with advanced colorectal cancer has a greater survival benefit. Patients with unresected primary lesions had poor efficacy using combination therapy. There were no statistically significant differences in PFS among gender, age, liver metastasis, lymph node metastasis, cetuximab use, and other factors (*p* > 0.05). We did not repeat impressive REGONIVO results. On the one hand, REGONIVO was an Ib phase study. On the other hand, the patients recruited usually had a better general situation and smaller tumor volume.

The most common adverse reactions to immunotherapy included fatigue, rash, pruritus, and diarrhea. The most frequent adverse reactions above grade 3 were anemia, elevated aspartate aminotransferase, and fatigue [18]. Regorafenib treated patients with some adverse reactions, such as hand-foot syndrome, fatigue, diarrhea, and hypertension [3, 19]. In this study, the adverse effects of combined treatment were mainly hand-foot syndrome, liver dysfunction, hypothyroidism, fever, fatigue, leukopenia, hypertension, platelet count decreased, diarrhea, and myocarditis. The incidence of grade 3/4 adverse reactions was 9.09%. Most of them are grade 1 or 2 reactions and can be tolerated after symptomatic treatment. One patient stopped treatment due to myocarditis and gradually improved after receiving high-dose cortisone therapy.

Immunotherapy is expensive and has the risk of adverse reactions, so it is very important to explore and make full use of biomarkers to predict the efficacy of immunotherapy in MSS colorectal cancer. Tumor mutation burden (TMB) has been an independent predictor of immunotherapy in multiple solid tumors. In MSI-H tumors, TMB is significantly higher than MSS tumors [20]. Therefore, whether TMB can be used as an independent predictor of colorectal cancer efficacy is still controversial. PD-L1 expression is considered to be one of the predictors of immunotherapy in lung cancer, gastric cancer, and other cancers [21]. Still, its predictive value in MSS colorectal cancer is currently believed to be limited [22]. Previous studies have shown that circulating tumor DNA (ctDNA) levels can identify patients who benefit from ICIs. An investigation had suggested that changes in ctDNA can predict the outcome of immunotherapy in MSS colorectal cancer patients [11]. Immune score, as a direct indicator of the internal immune status of the tumor, is also expected to be a predictor of the efficacy of MSS immunotherapy for colorectal cancer. We have not evaluated TMB, PD-L1 expression, immune score, and ctDNA in our cohort. The study offers a new option for third-line and later colorectal cancer patients. Additional analysis is needed to explore effective biomarkers on treatment outcome with this combined therapy.

In summary, regorafenib combined with toripalimab has demonstrated encouraging antitumor activity and acceptable tolerance in the third-line and beyond treatment of advanced colorectal cancer. This study is a small sample study in a single center. Although certain clinical results have been obtained, more profound studies on the combination are still needed to get better results for more patients with advanced colorectal cancer worldwide.

## Figures and Tables

**Table 1:** Clinical information of 33 advanced colorectal cancer patients (*n* = 33).

Characteristics	Statistics
Sex (male/female)	15/18
Age (years)	53.64 ± 10.34
ECOG (0/1)	10/23
Primary tumor location (left/right)	23/10
Liver metastases (yes/no)	20/13
Lung metastases (yes/no)	28/5
Lymph node metastasis (yes/no)	22/11
Excision of primary lesion (yes/no)	29/4
Number of previous chemotherapy lines	
(2/3 lines or above)	16/17
Regorafenib (80 mg/120 mg)	23/10
KRAS genetic testing (yes/no)	21/12
KRAS (wild/mutation)	13/8

Previous chemotherapeutic drug	
Fluorouracil (yes/no)	33/0
Oxaliplatin (yes/no)	33/0
Irinotecan (yes/no)	33/0
Raltitrexed (yes/no)	12/21

Previously targeted drugs	
Bevacizumab (yes/no)	28/5
Cetuximab (yes/no)	11/22

ECOG, Eastern Cooperative Oncology Group performance status.

**Table 2:** The efficacy was evaluated at the first follow-up (RECIST1.1).

Evaluation	*n* (%)
CR	0 (0)
PR	4 (12.12)
SD	12 (36.36)
PD	17 (51.52)
DCR	16 (48.48)
ORR	4 (12.12)

CR, complete response; PR, partial response; SD, stable disease; PD, progressive disease; DCR, disease control rate; ORR, objective response rate.

**Table 3:** Univariate analysis of clinical features and the curative effect of 33 advanced colorectal cancer patients (*n* = 33).

Characteristics		Total, no.	Effective, no. (%)	*P*
Sex	Male	15	9 (60)	0.323
	Female	18	7 (38.89)	

Age	<55 years	17	8 (47.06)	0.971
	≥55 years	16	8 (50)	

ECOG	0	10	8 (80)	0.034
	1	23	8 (34.78)	

Primary tumor location	Left	23	9 (39.13)	0.164
	Right	10	7 (70)	

Liver metastases	Yes	20	10 (50)	0.782
	No	13	6 (46.15)	

Lung metastases	Yes	28	11 (39.29)	0.032
	No	5	5 (100)	

Lymph node metastasis	Yes	22	12 (54.55)	0.286
	No	11	4 (36.36)	

Excision of primary lesion	Yes	29	15 (51.72)	0.181
	No	4	1 (25)	

Raltitrexed	Yes	12	4 (33.33)	0.2
	No	21	12 (57.14)	

Bevacizumab	Yes	28	11 (39.29)	0.034
	No	5	5 (100)	

Cetuximab	Yes	11	4 (6.36)	0.101
	No	22	12 (54.55)	

Number of previous chemotherapy lines	2 line	16	12 (75)	0.005
	3 lines or above	17	4 (23.53)	

Regorafenib	80 mg	23	10 (43.48)	0.456
	120 mg	10	6 (60)	

KRAS	Mutation	8	5 (62.5)	0.203
	Wild	13	4 (30.77)	

ECOG, Eastern Cooperative Oncology Group performance status.

**Table 4:** Multivariate Cox regression analysis of PFS of 33 advanced colorectal cancer patients.

Characteristics	HR	95% CI	*P*
Number of previous chemotherapy lines	5.941	1.765–19.991	0.004
Excision of primary lesion	0.215	0.54–0.853	0.029
ECOG	—	—	0.364
Primary tumor location	—	—	0.753
Bevacizumab	—	—	0.087
Lung metastases	—	—	0.111

ECOG, Eastern Cooperative Oncology Group performance status.

**Table 5:** Adverse reaction of 33 advanced CRC patients treated with regorafenib and toripalimab as third-line or above.

Adverse reaction	Grade (*n*)				Proportion (%)	
	1	2	3	4	Grades1–4	Grade3/4
Fatigue	5	2	0	0	21.21	0
Hypertension	3	1	0	0	12.12	0
Hypothyroidism	7	1	0	0	24.24	0
Hand-foot syndrome	8	2	1	0	33.33	3.03
Leukopenia	5	0	0	0	15.15	0
Platelet count decreased	1	1	0	0	6.06	0
Myocarditis	0	0	0	1	3.03	3.03
Liver dysfunction	7	1	1	0	27.27	3.03
Fever	6	2	0	0	24.24	0
Diarrhea	0	1	0	0	3.03	0

## Data Availability

The datasets used and/or analyzed in the present study are available from the corresponding author upon request.
